# Image Registration Improves Confidence and Accuracy of Image Interpretation

**Published:** 2007-05-12

**Authors:** Bradley J Erickson, Julia Patriarche, Christopher Wood, Norbert Campeau, E. Paul Lindell, Vladimir Savcenko, Norman Arslanlar, Liqin Wang

**Affiliations:** Department of Radiology, Mayo Clinic, Rochester, MN

## Introduction

Sequential MR examinations of the brain are the primary method for clinical as well as research assessment of the effects of therapy on brain tumors. In clinical practice, visual comparison is the primary method of assessing changes that indicate tumor response or progression. This is a labor-intensive process involving visual search for changes between examinations on multiple images from multiple image types. Furthermore, some of the changes that may be perceived could be do to acquisition-related changes, rather than changes in the tumor status. One of these changes is the change in the patient position between the two time points. While every effort is made to acquire images in the same plane as prior exams, this is rarely achieved. In this study, we evaluated computerized image registration (A.K.A. image alignment) on accuracy and confidence.

## Methods

### Study selection

After IRB approval, we collected a series of 100 sequential MRI examination pairs in patients with primary brain gliomas in which there had been no intervening surgery. Furthermore, we selected those in which the clinical radiologist interpretation indicated either subtle or no change in the tumor. The interval between examinations ranged from 35 days to 375 days, with the median being 75 days. Tumor types included astrocytoma, oligodendroglioma, and mixed oligo-astrocytomas, and tumor grade ranged from 2 to 4 on the World Health Organization scale.

Examinations consisted of 3 mm thick contiguous T1, T2, FLAIR, and T1-post contrast images obtained with a 1.5T GE Signa (GE Medical Systems, Waukesha, WI) scanner. The T1-weighted images were spin-echo sequences with TR ranging from 400 ms to 620 ms and TE min full. T2-weighted images were fast spin echo images TR 3500–4000 ms and TEeff of 120 ms. FLAIR images were also fast spin echo with TR 11000 ms, TI 2250 ms and TE 250 ms.

### Image processing

**Figure 1:**
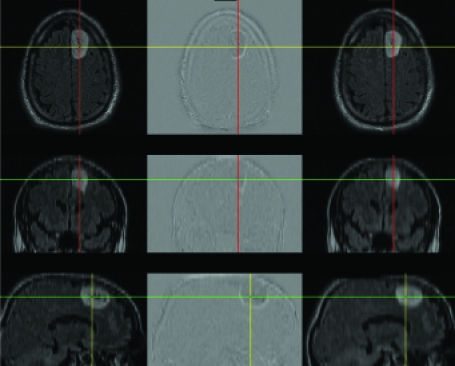
Verification of alignment was performed by visually assessing that linked cursors as well as subtraction images showed good alignment. In this case, most of the brain is even gray, while the outline of the tumor demonstrates interval change.

Examinations were anonymized, and an additional set of images was created in which the T1, T2, and FLAIR images of the most recent exam as well as all 4 series of the ‘old’ exam were registered to the current post-contrast series. Image registration was accomplished using the mutual information algorithm, as implemented within the Insight Toolkit (ITK) which is available at http://www.itk.org. The concepts of mutual information image registration have been described elsewhere; a recent comprehensive review nicely covers the topic (Pluim, Maintz et al.). Briefly, the mutual information metric is maximum when images that may have very different contrast properties are spatially aligned. This is possible because even with different contrast properties, the gray value of one image will best predict the gray value of another image when they are aligned. By iterating various geometric transformations, one can use the mutual information measurement to determine the optimal transformation to align images. The time to compute the registration was recorded as part of the process. After completion, satisfactory alignment was visually confirmed using a ‘flicker test’ as well as linked cross-hairs (Fig 1). For those series that did not align, interactive adjustment of algorithm parameters was performed to achieve satisfactory alignment.

### Ratings

Six neuroradiologists reviewed the images in two settings. In each setting, half the examinations were registered and the other half not (in a random order). If a given exam pair was presented in a registered form for the first setting, it was presented as non-registered in the second setting, and vice-versa. The raters assigned a rating to each exam pair on a scale from 1 to 20 where 1 meant absolute confidence of no progression of the tumor while 20 meant absolute confidence of progression. 10 would mean as likely to be progressing as not. For the purpose of this study, regression was ignored—only progression vs non-progression was considered. At least 1 month passed between the two sessions.

### Statistical methods

Later, the same group met and a consensus opinion was reached as to whether there was progression or not for each of the 100 exam pairs (during this session, they had access to subsequent exams as well as the clinical record). Agreement was reached in 97 of the 100 cases. In some cases, there were areas of both progression and regression, but any study showing progression in any area was considered progression. Ratings were entered into an Excel (Microsoft Corp, Redmond, WA) spreadsheet in which summary statistics were calculated. Data was also entered into ROCFIT, and ROC curves generated for each radiologist and a group ROC curve was created by averaging at each sensitivity value (McNeil and Hanley, 1984; Metz, 1986; Metz, 1989; Metz, Shen et al. 1990). These 97 cases represented the final test set, and the consensus was the ‘correct’ answer.

## Results

**Figure 4:**
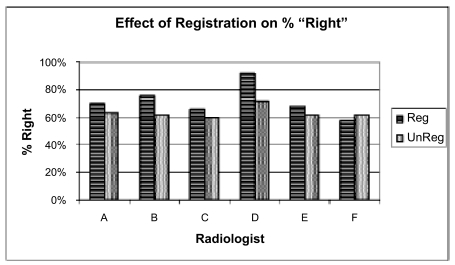
Readers more frequently got the ‘right’ answer when using registered images. The ‘right’ answer was the consensus of the panel convened after the readings were obtained. The group difference was statistically significant at the p < 0.03 level.

**Figure 5:**
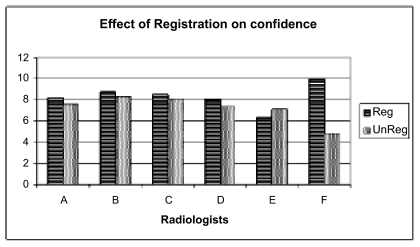
Readers were more confident of their conclusions when comparing registered examinations.

**Figure 6:**
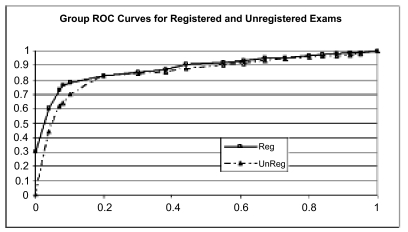
Group ROC curve for registered versus unregistered examinations. The A_z_ value for the registered exams was 0.87 vs 0.78 for unregistered examinations. Grouping of individuals was accomplished by averaging the performance of each radiologist at each sensitivity value.

We found that image registration improved accuracy for 5 of the 6 readers, and the difference was statistically significant at the p < 0.03 level (see Figure 4). There was also a higher confidence in ratings (Figure 5), though this was not statistically significant. Finally, there was a greater A_z_ value for the registered exams as well: 0.87 vs 0.78 (see Figure 6). Anecdotally, there was also consensus that image registration made comparison more efficient and introduced no significant image degradation.

Satisfactory alignment was achieved without user intervention in 678 out of 700 series (100 patients with 3 series from the current exam were matched to the post-contrast sequence, and all 4 series of the prior exam). Manual adjustment of tissue thresholds or adjustment of alignment starting position resulted in satisfactory alignment for 11 of the remaining 21 series. The last 10 series could not be satisfactorily aligned due to significant patient motion which caused substantial image degradation. These series were not available for the visual comparison, and the corresponding series were also deleted from the unregistered set to avoid unblinding. The average time to compute the registration was 58 seconds per series on a 2 Ghz Pentium™ computer with 1GB RAM.

## Discussion

It is now computationally tractable and feasible to automatically align old examinations to new. For typical studies, this can be accomplished in about 1 minute per series on desktop computers. The quality of alignment depends heavily on the quality and spatial resolution of the examinations—significant artifacts from patient motion, for example, will cause a poor alignment in most cases. Thick slices and large gaps will also reduce the accuracy of alignment—4 mm thick slices with no gap is a reasonable upper limit. But if a good quality examination is performed, the quality of interpretation, as measured by accuracy of identifying progression or stability, can be improved by retrospective image alignment. This is most noticeable in cases of subtle progression, which is more common with shorter scan intervals.

The cases selected for this study were selected by finding those in which there was either subtle change or no change. In cases where there is gross progression, registration is unlikely to make it more obvious. However, for subtle disease, precisely aligning studies can help to clarify if apparent changes are due to variations in patient alignment or true disease state changes. Furthermore, even in cases of gross change, registration may help to identify subtle regional changes that are of clinical significance.

Some have described a process for acquiring the MRI images in an aligned condition (Gedat, Braun et al.; van der Kouwe, Benner et al. 2005). This is likely to produce similar benefits. However, we do not believe it will replace the need for retrospective alignment. First, this procedure uses a relatively low resolution scan, which in turn, limits the accuracy of the registration. Such a scan will clearly be better than the current process, but is unlikely to achieve the subvoxel resolution, particularly as voxels become less than 1mm in all dimensions. In addition, patients often move between series. Doing the alignment procedure at the start of the exam would not address these changes. We believe acquiring aligned scans is a complementary procedure—reducing the misregistration will reduce the interpolation artifacts. But it is unlikely to ever be as accurate as post-acquisition registration between high resolution scans.

We and others have demonstrated improvements in inter-rater agreement as well as efficiency gains associated with automated alignment (Schellingerhout, Lev et al. 2003; Lau, Erickson et al. 2004; Erickson, Andriole et al. 2005). This should not be surprising, given that requiring the radiologist to mentally ‘re-slice’ the image both requires time and effort, and will often introduce an element of uncertainty. This suggests that image alignment has nearly all positive effects for interpretation—it can make readers more accurate and more efficient. This is truer as data sets become larger due to thinner slices.

Precise alignment is also a requisite for nearly every multiparametric image processing technique such as segmentation and classification We (Patriarche and Erickson) have described the use of precisely registered examinations for semi-automated detection of changes in brain tumors.

We did not study issues of how the images should be optimally presented to a rater. We presented both registered and unregistered images in the traditional side-by-side fashion. As can be seen from Figure 1, subtraction of the images can highlight interval changes as well, and may be more effective in conveying differences. We view this, as well as ‘in-place’ or flicker mode display option, as an important future direction of research on how to optimally interpret images. However, until that work establishes incremental value, and until such processing and display option is available in the image display workstation, side-by-side display will be the norm.

## Conclusion

Future clinical studies using imaging as an endpoint should use image registration to improve accuracy and efficiency of image interpretation. This can be accomplished with widely available computer algorithms. Displaying the images side-by-side is acceptable, though future research may demonstrate alternative display schemes that further increase the advantages image registration.

## Figures and Tables

**Figure 2:**
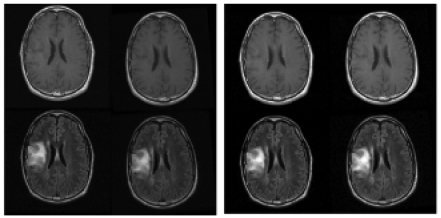
Example slice from a case which was judged stable. Fig 2a. shows how the images were presented if the registered pair was presented, and Fig 2b shows the same exam after registration. For the study, raters had access to all relevant images, not just a single slice as shown here. These images were acquired with good alignment, and so no improvement was shown in this case for any rater.

**Figure 3:**
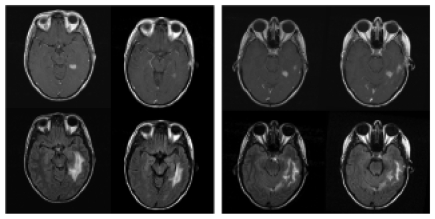
Example slice from a case which was judged to be progressing. Fig 1a. shows how the images were presented if the registered pair was presented, and Fig 1b shows the same exam after registration. For the study, raters had access to all relevant images, not just a single slice as shown here. There is mild to moderate misregistration in the original images.
